# Isocitrate Dehydrogenase 2 (IDH2)-Mutant Metastatic Esthesioneuroblastoma: A Case Report and Review of Current Therapeutic Options

**DOI:** 10.7759/cureus.78618

**Published:** 2025-02-06

**Authors:** Rita Banha, Rute Fernandes, Charalampos S Floudas, Claudia Vieira

**Affiliations:** 1 Medical Oncology, Hospital Do Divino Espírito Santo, Ponta Delgada, PRT; 2 Medical Oncology, Instituto Português De Oncologia, Porto, PRT; 3 Center for Immuno-Oncology, Center for Cancer Research, National Cancer Institute, National Institute of Health, Bethesda, USA

**Keywords:** esthesioneuroblastoma, idh2 mutation, metastasis, olfactory neuroblastoma, targeted therapy

## Abstract

Esthesioneuroblastoma (ENB) is a rare malignant neoplasm of the nasal cavity. The clinical course is heterogeneous and, currently, there is no consensus regarding the correct management of this disease. We present the case of a 37-year-old man with a diagnosis of an ENB, progressing, two years after radical treatment with surgery and chemoradiotherapy, with spinal and leptomeningeal metastasis. He underwent palliative radiotherapy directed to the sacral thecal sac lesion and chemotherapy with platinum and etoposide, however, with disease progression. Next-generation sequencing (NGS) revealed an isocitrate dehydrogenase 2 (IDH2) mutation. Thus, an effort was made to initiate therapy targeting the mutation found. Given that chemotherapy has a limited role in the advanced disease setting, we aim to discuss novel treatment options, such as targeted therapy.

## Introduction

Esthesioneuroblastoma (ENB), also known as olfactory neuroblastoma, is a rare malignant neoplasm of the nasal cavity arising from the olfactory neuroepithelium. It accounts for about 3%-6% of nasal cavity neoplasms [1,2].

Diagnosis is often complicated and delayed by non-specific symptoms. The clinical presentation will depend on the local or regional extent of the tumor, but signs such as nasal obstruction, epistaxis, anosmia, or rhinorrhea are usually present [2,3]. Evaluation involves performing a head computer tomography (CT) or magnetic resonance imaging (MRI) to assess the local extent of the disease. Staging is done with full-body CT or positron emission tomography (PET 18F-FDG). The most used staging system is the Kadish system, which has been modified by Morita et al. and classifies the primary tumor based on the extent of local tumor anatomic involvement [4]. Biopsy is mandatory, and histological classification according to the Hyams et al. grading system seems to have a correlation with survival outcomes [5].

The clinical course of ENB is heterogeneous, ranging from indolent growth tumors to aggressive behavior with local and distant metastasis. For this reason, and given its rarity, there is currently no consensus regarding management [6]. Treatment for resectable disease involves surgery, radiotherapy, and/or chemotherapy [1,2]. The clinical course after that varies, with recurrence rates of approximately 30% [6]. When metastasis occurs, cervical lymph nodes are the most frequent site [6,7]. Distant metastasis is less frequent, but metastasis to the brain or spine has been described, and leptomeningeal involvement is associated with a poor prognosis [7].

The role of systemic chemotherapy in the recurrent or progressive disease setting is limited, so further research with targeted therapies is needed and can provide additional treatment options [8]. Some successful cases with the use of target therapies have already been reported [9]. The authors report a clinical case of an ENB that progressed two years after radical treatment with surgery and chemoradiotherapy, with spinal and leptomeningeal metastasis. We aim to discuss current therapeutic options and the role of next-generation sequencing (NGS) in defining further lines of treatment in polytreated metastatic cases.

## Case presentation

A 37-year-old man with a history of smoking and no known comorbidities or family history of cancer developed pain and a sensation of pressure on the left hemiface in 2020. He was referred to an otorhinolaryngologist, where evidence of a neoformation in the left nasal fossa was found on rhinoscopy.

He underwent endonasal microsurgery with excision of the neoformation. Pathology revealed a high-grade ENB, Grade III/IV according to the Hyams Grading System. After surgery, an MRI and PET 18F-FDG were performed, showing evidence of tumor persistence/recurrence in the left nasal fossa. No regional or distant metastasis was detected. A new intervention was performed with excision of the ENB via nasal-sinus endoscopic surgery. The pathology report confirmed a Grade III (Hyams Grading System) ENB with neoplastic extension beyond the nasal cavity and paranasal sinuses.

After discussion in a multidisciplinary tumor board, it was decided to perform complementary chemoradiotherapy. The primary tumor area (based on pre-surgery imaging) was irradiated with 66 Gy, the residual tumor area (based on post-surgery imaging), including the bilateral ethmoid, bilateral nasal cavities, sphenoid sinus, and left jaw, received 60 Gy, and the right maxillary sinus, frontal sinus, and cervical, retropharyngeal, and bilateral levels I-II lymph nodes were irradiated with 54 Gy. The treatment was delivered in 33 fractions of 2 Gy/day, five days a week, using the volumetric modulated arc therapy (VMAT) technique. Concomitantly, the patient received chemotherapy. Initially, chemotherapy with cisplatin (100 mg/m² every 21 days) was administered; however, shortly after the first cycle, the patient began experiencing complaints of bilateral tinnitus. Therefore, the regimen was changed to carboplatin (AUC 1.5). This regimen was also poorly tolerated, with significant complaints of anorexia and emesis. Chemotherapy was then terminated after two cycles of carboplatin.

**Figure 1:**
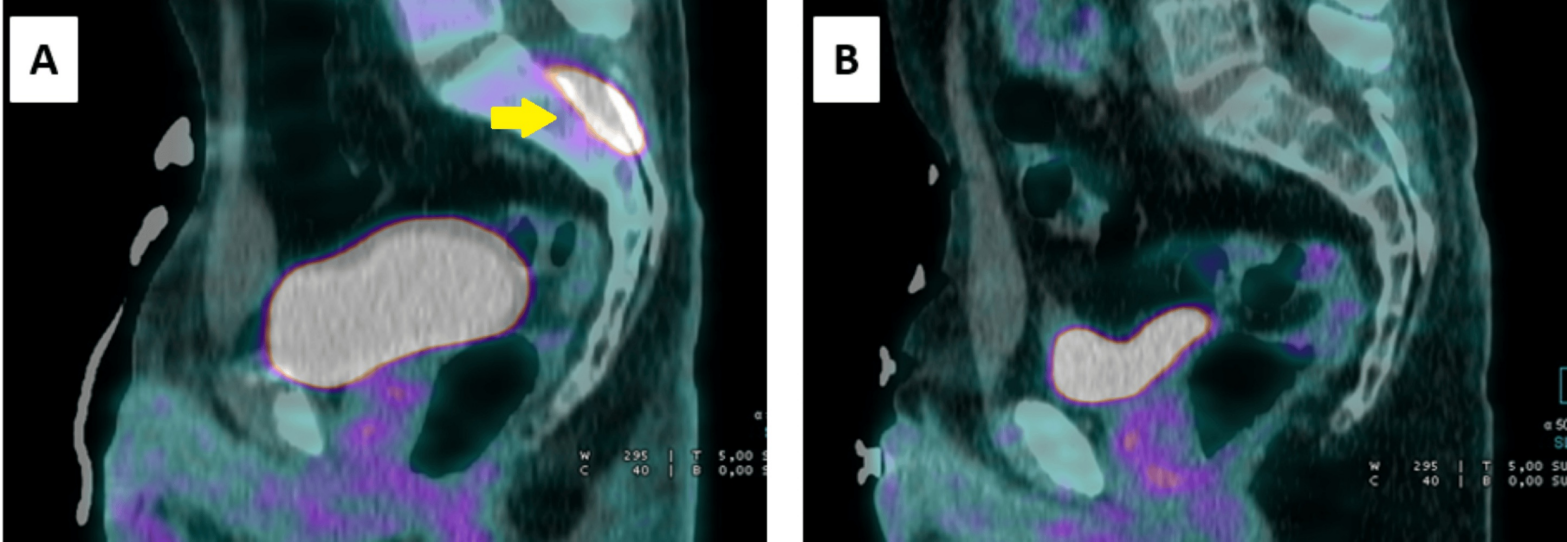
A) PET at the diagnosis of metastatic disease, showing a lesion in the intradural topography at the sacral level (yellow arrow). B) PET after six cycles of chemotherapy, showing a complete response to the administered therapy. PET, positron emission tomography

Frequent clinical and imaging surveillance with brain MRI was performed, with no evidence of local recurrence. However, approximately 23 months after the end of chemoradiotherapy, the patient began experiencing complaints of sacrococcygeal pain. An MRI revealed sacral root metastasis. PET 18F-FDG confirmed lesions in the intradural topography at the lumbar and sacral levels and further revealed bilateral cervical and supraclavicular lymph node metastasis (Figure 1A).

The case was once again reviewed by a multidisciplinary tumor board, resulting in a decision to proceed with systemic treatment and palliative radiotherapy. The patient underwent radiotherapy directed at the sacral thecal sac lesion (20 Gy in 5 fractions of 4 Gy/day), leading to an improvement in pain. Palliative chemotherapy with cisplatin and etoposide was initiated (cisplatin 80 mg/m² on day one, and etoposide 100 mg/m² on days one, two, and three, every 21 days). The patient was also referred to a precision oncology program. A PET 18F-FDG scan after three cycles showed evidence of a complete response to the chemotherapy. Due to complaints of decreased hearing acuity after the fourth cycle, which was confirmed by an audiogram, the regimen was changed to carboplatin (AUC 5) and etoposide. The patient completed a total of six cycles of treatment with reasonable tolerance. A follow-up PET scan continued to show evidence of a complete response, and it was decided to proceed with surveillance (Figure 1B).

**Figure 2:**
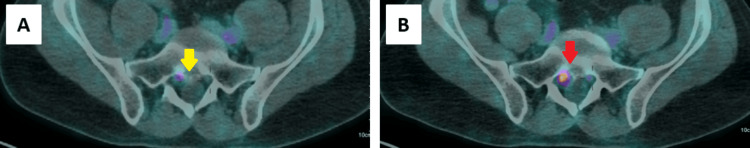
Evidence of worsening metabolic hyperactivity at the medullary/intracanal level, from image A (before resuming chemotherapy - yellow arrow) to image B (after chemotherapy - red arrow).

Three months after the last cycle of chemotherapy, the patient developed complaints of diplopia. Following an assessment by Neurology, the performed exams confirmed leptomeningeal tumor dissemination, without any structural brain lesions. It was decided to resume chemotherapy with carboplatin and etoposide. The patient experienced significant clinical benefit from the resumption of treatment, but with hematological toxicity, which required dose adjustments (carboplatin AUC 4 and etoposide 75 mg/m²). The best response obtained was stable disease; however, disease progression was observed at the medullary/intracanal cervico-dorso-lumbo-sacral level after seven cycles of treatment (Figure 2A and Figure 2B).

NGS revealed an isocitrate dehydrogenase 2 (IDH2) mutation. Taking this information into account, along with the fact that the patient maintained a good performance status, an attempt was made to include the patient in a clinical trial or to access therapy targeted at the IDH2 mutation, such as enasidenib or vorasidenib. However, neither of these options was available at that time, so it was decided to initiate a new line of palliative chemotherapy with paclitaxel. The patient completed a total of eight cycles of weekly paclitaxel with reasonable tolerance, but unfortunately, new evidence of disease progression emerged. At this point, the patient was enrolled in a clinical trial for an agent targeting the IDH2 mutation. However, shortly after treatment initiation, his general condition worsened, including the development of severe hyponatremia, which required inpatient management, followed by re-admission for infectious complications. The patient ultimately passed away during the second hospitalization, despite the measures taken, approximately 49 months after the initial diagnosis.

## Discussion

ENB is a rare malignant neoplasm of the nasal cavity with no defined treatment regimens. For localized diseases, multimodal treatments (surgery, radiotherapy, and chemotherapy) are frequently chosen [1,2]. Given its rarity, there is limited evidence regarding the management of advanced and metastatic disease. In a systematic review and meta-analysis by Marinelli JP et al., 12% of patients developed distant metastatic disease following initial definitive curative treatment of the primary tumor [8]. The median time to the development of distant metastatic disease was 15 months, and the most common metastatic sites were bone, drop spinal metastasis, and lungs [8]. Our patient presented with distant sacral root metastasis approximately 23 months after completing radical treatment but also had regional lymph node metastasis at the same time.

Regarding the treatment of advanced and metastatic disease, no standard therapeutic protocol exists, and the role of chemotherapy appears to be limited [2,8]. Platinum- and etoposide-containing regimens are the most frequently reported, but responses are typically not long-lasting [10,11]. Non-platinum regimens, such as those with irinotecan and docetaxel, have also been reported [12]. Temozolomide may be considered, as it has been used successfully in a patient with intracranial metastatic ENB [13].

In addition to the lack of consensus regarding chemotherapy, some patients may not tolerate multiple courses of cytotoxic therapy, as seen in the patient described here. For this reason, NGS can play an important role in providing a molecular rationale for targeted therapy. Gay LM et al. reported that, in 41 relapsed or refractory ENBs, 51% of tumors exhibited clinically relevant genomic alterations [9]. Specifically, potentially targetable alterations were identified in genes associated with the PI3K/mTOR pathway in 11 samples, and in the cyclin-dependent kinase cell-cycle regulatory pathway in six samples, with everolimus and palbociclib mentioned as potential therapies [9].

Several case reports have described the successful use of targeted therapy. Spengler M et al. reported a 69-year-old woman with heavily pretreated metastatic ENB harboring a fumarate hydratase mutation, who achieved a partial response to pazopanib for at least four years [14]. Preusser et al. reported on a 69-year-old man with recurrent ENB, who achieved disease stabilization for 15 months with the tyrosine kinase inhibitor sunitinib, dying thereafter due to trauma and without evidence of disease progression [15].

Regarding IDH2 mutations, Wu L et al. reported that tumors carrying these mutations represent a subset with aggressive behavior and poor prognosis [16]. Furthermore, Wu L et al. suggested the value of a molecular classification based on IDH2 mutations, considering that ENB is a heterogeneous disease, with the current classification based on histological criteria [16]. Indeed, other groups have also proposed a classification based on molecular subtypes [17]. A phase II clinical trial evaluating the use of enasidenib in IDH2-mutated malignant sinonasal and skull base tumors, including olfactory neuroblastoma, was recently initiated, and the results will be relevant to cases like the one reported here [18]. Although there are ongoing clinical trials, we are not aware of any case reports of patients with ENB treated with IDH2 inhibitors. Given their role in the treatment of other neoplasms, such as gliomas, their use in ENB patients should be explored.

Another therapeutic option that has been reported for the treatment of unresectable recurrent or metastatic ENB is peptide receptor radionuclide therapy (PRRT). Hasan OK et al. reported, in a cohort of seven patients, the use of PRRT based on the overexpression of cell surface somatostatin receptors (SSR) on SSR imaging, with favorable clinical and imaging responses: four patients had disease response, and two had disease stabilization [19].

We present a summary of the therapeutic options, other than platinum-based chemotherapy, for the management of advanced and metastatic ENB in Table 1.

**Table 1:** Therapeutic options discussed for the management of advanced and metastatic ENB. ENB, esthesioneuroblastoma

Reference	Treatment(s) for advanced or metastatic disease	Number of patients	Outcomes
Kiyota N et al. [12]	Irinotecan plus docetaxel	12	Partial response in 3 patients.
Wick W et al. [13]	Temozolomide	1	Stable disease for 23 months.
Gay LM et al. [9]	Everolimus	1	Stable disease for 12 months.
Spengler M et al. [14]	Pazopanib	1	Partial response for 48 months.
Preusser et al. [15]	Sunitinib	1	Stable disease for 15 months.
Hasan OK et al. [19]	Peptide receptor radionuclide therapy	7	Disease response in 4 patients; disease stabilization in 2.

## Conclusions

ENB is a rare disease, with no currently well-defined treatments in recurrent and metastatic settings. NGS can help identify promising new therapeutic options that may be adopted into clinical practice. Furthermore, enrollment in clinical trials should be encouraged whenever possible.

We believe that case reports are valuable for the real-world management of rare neoplasms. Additionally, efforts must be made to standardize treatment regimens for this and other uncommon malignancies.
